# Simulations to Teach Science Subjects: Connections Among Students’ Engagement, Self-Confidence, Satisfaction, and Learning Styles

**DOI:** 10.1007/s10639-022-10940-w

**Published:** 2022-02-08

**Authors:** Firas Almasri

**Affiliations:** 1 Centre for Education Studies, University of Warwick, Coventry, CV4 7AL UK; 2 Present Address: Department of Mathematics and Natural Sciences, Gulf University for Science and Technology, Hawally, Kuwait

**Keywords:** Simulations, Science Education, Engagement, Self-confidence, VAK learning styles

## Abstract

With the increasing technology integration practices in education, the adoption of computer simulations to teach conceptual understanding of science concepts is widely accepted by educators across the globe. To understand the connections between learners’ engagement and satisfaction with simulations for science learning and their learning styles, the present study analyzed 1034 university students' perceptions and experiences of using simulations for learning physics, chemistry, and biology subjects. The study took place in a large public university in a gulf country. Precisely, this study provides an empirically driven exploration of the connection between tertiary students' engagement and satisfaction with simulation-based learning and their learning styles. The findings of this study showed that the participants showed a very high level of engagement and satisfaction with the use of simulations for learning science concepts in the subjects of physics, chemistry, and biology. Their self-confidence and VAK learning styles, particularly the kinesthetic style, were significant predictors of their engagement and satisfaction with the learning process. The findings from this study have implications for the benefit of researchers and practitioners interested in the effective adoption of computer simulations as a pedagogical approach in science education.

During the last few decades, educational practices have adapted to the new technological scenario by taking advantage of the benefits that emerging technologies provide (Matute-Vallejo & Melero-Polo, 2019). Within this situation, numerous advanced educational techniques have freshly appeared to enhance teaching-learning practices. One of such pedagogical tools is the use of computer simulations to enhance teaching and learning processes (Vlachopoulos & Makri, 2017). Computer simulations are the programs that allow learners to systematically explore hypothetical situations, interact with that situation, and explore the changes occurring to the situation as a result of their interaction. Recent studies reveal that the use of simulation results in effective and plentiful learning gains (Blake & Scanlon, 2007; Matute-Vallejo & Melero-Polo, 2019; Rutten et al., 2012; Sanina et al., 2020).

Simulations are used in a variety of settings, including in education, aviation, nursing, social work, and transport (Rooney & Nyström, 2018). The use of simulations for science learning is currently a widespread practice (Langbeheim & Levy, 2019; Rooney & Nyström, 2018, Rutten et al., 2012). Their adoption in teaching science topics has the potential to result in higher learning improvements in ways not previously possible (Akpan, 2001). Further, they have secured a room in the classroom as strong accompaniments to the repertoire of teachers, either as a supplementary element to existing teaching approaches or as a substituting part of the curriculum (Rutten et al., 2012).

Regardless of the type of pedagogical techniques used, students' engagement and satisfaction with the learning process are of utmost importance. Learners’ engagement is the energy and effort that they devote while interacting with the learning process (Bond, 2020; Khlaif et al., 2021). It basically shows the level of students' involvement with the learning environment. Positive and effective learning gains are almost impossible to be achieved if students' are not actively engaged with and feel displeased with the learning environment. Previous research endorses learning style as an influencer to the learning environment (Altun & Serin, 2019; Azzi et al., 2020; Murray, 2004; Oxford et al., 1991; Rooney, 2018). Students' are more engaged and perform better if the learning process matches their learning style preferences (Altun & Serin, 2019). Exclusively, in an E-learning environment, the learning style has a larger influence on the performance of the learners as well as in the design of the E-learning environments (Deborah et al., 2014).

In exploring the use of computer simulations for science learning, the intent of this study is not only to document the tertiary students' engagement and satisfaction with the learning process, but the study also seeks to examine the potential role of students' preferred learning style on such learning environment. While several studies have been conducted in the area of using simulations for science teaching (Blake & Scanlon, 2007; Falloon, 2019), there is not sufficient literature that provides information on the role of students' learning styles i.e., auditory, visual, and kinesthetic in their engagement, self-confidence, and satisfaction with simulated-supported learning activities for science courses. The present study is important, given the acceptability/popularity of simulation-based teaching at various educational settings, as it examines the role that learning styles may play in explaining learners’ engagement and satisfaction with the simulation-based learning process.

## Literature Review

The emerging technologies of the current time, particularly the dynamic characteristics of the Internet, offer several avenues for innovative pedagogies to support effective learning. Most importantly, these emerging technologies are credited with transforming learning into an active and engaging process (Soomro et al., 2020; Vlachopoulos & Makri, 2017). Computer simulations, one of the widely used forms of technology integration into the teaching-learning process (Vlachopoulos & Makri, 2017), support modeling-based learning, allowing the participants to generate questions and to construct, test, and evaluate the theoretical models (Lee et al., 2021). The section ahead presents a review that starts with the accepted definitions of computer simulations and then moves towards its use in teaching particularly to enhance students'conceptual understanding of science topics. The section then concludes with an overview of learning styles and the potential role they can play in students'engagement and satisfaction with simulation-based teaching in science courses.

### What is a Simulation?

The existing literature refers to the term simulation in different words. It is generally defined as the imitation of a process or situation (Rooney, 2018). They are also referred to as computational representations of real or hypothesized situations or phenomena and an environment that provides a dynamic, interactive, and visualized learning experience (Fallon, 2019). The idea of computer simulations has also been connected to a web-based or remote laboratory, which provides participants a way to conduct real-world experiments at a distance (Tho & Yeung, 2016). Computer simulations have a great application in a variety of fields, including education, aviation, and health sciences. They empower participants to grow their skills without being exposed to a situation that may result in severe consequences or require extra-high expenses otherwise. More importantly, such computer programs offer an efficient way of monitoring experimental variables, giving an opportunity for exploration and hypothesizing (Blake & Scanlon, 2007).

Blake and Scanlon (2007) describe computer simulations as programs that contain a representation of an authentic system or phenomenon that participants study through interaction with the simulation. In addition to embracing the capability to standardize the educational experiences of the growing student cohorts (Rooney, 2018), computer simulations allow simultaneous representations of the real and theoretical behaviors of a phenomenon and provide visualization of choices and consequent effects (Blake & Scanlon, 2007). Visualization in computer simulations facilitates learners to grasp real-world data through multiple representations (Goldstone & Son, 2005; Lee et al., 2021; Rutten et al., 2012). Accordingly, such excellent features of computer simulations offer students' a straight experience of the (simulated) world. Taking into consideration the above-mentioned definitions and descriptions of ‘simulation’, we tend to follow the simple and brief definition given by Vlachopoulos and Makri (2017) in this paper. According to them, “simulations create a scenario-based environment, where students' interact to apply previous knowledge and practical skills to real-world problems” (Vlachopoulos & Makri, 2017, p.4).

### The Use of Computer Simulations in Teaching

Computer simulation emphasizes the learner as an active agent to construct their knowledge. They have extraordinary potential to facilitate authentic inquiry practices that include articulating questions, hypothesis formulation, exploring the effect of data in a situation, and theory revision (Rutten et al., 2012). Wang et al. (2010) also highlight the value of simulations as they let learners effortlessly manipulate objects (virtual manipulatives), generate novel representations, carry out experiments to test hypotheses and tentative ideas.

Previous research indicates that simulations have a constructive influence on learning goals (Vlachopoulos & Makri, 2017). Simulation-based learning not only boosts students' engagement and motivation to construct new knowledge but also improves participants’ comprehension of the topics being learned at the cognitive level. This kind of learning has cognitive affordances to facilitate personal experimentation and offers avenues for making connections to existing knowledge (Lindgren et al., 2016). Lee et al. (2021) documented that the cognitive dimension is the most noticeable and is facilitated by the perceived usefulness and usability of the computer simulations for teaching. They argue such computer programs could enhance students' learning in the cognitive and affective domains and improve students' satisfaction and engagement with the learning process. According to Wang et al. (2010), simulations are also helpful in developing mindfulness of personal thinking processes (metacognition).

These days, it is common to see simulation laboratories in modern higher education institutions particularly to support nursing, health, and medical education. Such in-between spaces are mostly prefigured as providing health professionals with learning practices that link professional education and work (Rooney, 2018). For example, in an attempt to examine the impact of patient simulation, Shin et al. (2015), through a meta-analysis, found significant post-experiment gains in various domains for students' who had experienced simulation-based training in comparison to the students' in the control group. The findings led them to conclude that simulations are more effective than conventional pedagogical approaches. By encouraging learners to apply their knowledge to real-world problems using a scenario-based technique, simulation-based teaching is recognized to help develop students' analytical, critical thinking, strategic, and problem-solving skills (Robertson et al., 2009; Sanina et al., 2020).

### Computer Simulations in Conceptual Understanding of Science Topics

Given that computer simulations are now widely available for an extensive range of science subjects, they have become an essential part of several science curricula (Rutten et al., 2012). Researchers have sufficiently attempted to see the effectiveness of simulations for the teaching of conceptual understanding of science topics (Lee et al., 2021). Based on an experimental study involving primary school students' in New Zealand, Fallon’s (2019) concluded simulations can be valuable for teaching simple physical science concepts, and offering students' the openings to be involved in higher-order thinking processes. Lee et al. (2021) noted that this pedagogical approach is a great way to develop learners’ attitudes toward science topics, including optical lenses, moon phases, kinetic modular theory, trajectory motion, and electromagnetism. While using simulations in science is not new, adopting simulations for science teaching in the context of middle east countries is not sufficiently reported in the literature.

### Student Engagement and Satisfaction with the Learning Process

The term student engagement is described as “how involved or interested students appear to be in their learning” (Axelson & Flick, 2010, p. 1). According to Chapman (2003), it refers to learners’ cognitive investment in active participation and emotional commitment to their learning. It is considered to be causally related to learning and student success. For this specific study, student engagement refers to the degree of cognitive and perceptual involvement learners feel in their experience with simulation-based learning activities (Lindgren et al., 2016). Student satisfaction with the learning environment is another construct that directly relates to student learning. It basically refers to a gratifying emotional state stemming from an individual’s performance of the role of being a student and encourages engagement within the learning environment (Pelletier et al., 2016). Keeping in view the significance of both student engagement and satisfaction, it is implied that a learning activity including the one facilitated by computer-based simulations may not be effective to achieve the desired results unless students' are not only engaged with the process but are also satisfied with the learning environment.

### Learning Styles

The studies in psychology signify that people have several individual differences in decision-making, problem-solving, and learning processes (Fatahi et al., 2016). While working towards establishing effective and efficient learning experiences for the learners, educators and instructional designers need to consider learners’ differences. When students' are taught as per their abilities, interests, and desires, larger and effective learning gains can be achieved (Altun & Serin, 2019). One of the most important individual differences that directly influence students' learning experiences is their learning styles. Learning style is defined as a method that a learner prefers and prioritizes in the learning process (Khamparia & Pandey, 2020; Kolb, 1984). For example, an individual learner might prefer and learn a topic better by hearing a lecture than by reading the same concept. On the other hand, an individual having decent visual memory and weak verbal ability may enjoy and learn more from the content presented visually.

Learning style is one of the multifaceted factors in the learning environment influencing the learner’s motivation and capability to learn effectively (Murray, 2004). It reflects the cognitive, affective, and physiological expressions of individuals in observing, and interacting with the learning situation. Students' learn in different ways, especially incorporating and processing different types of information in distinct manners. There are several learning styles assessment models such as Kolb learning style indicator (experiential) (Kolb, 1984), Gregorc style delineator (cognitive) (Gregorc, 1985), Felder–Silverman Index of learning styles (psychological) (Felder & Silverman, 1988) and Fleming VAK model (meta-learning theory model) (Fleming, 2001).

Most of the contemporary learning style theories have attentive on either the cognitive learning style, such as the holistic/analytical approach to learning, or the perceptual learning style, such as the visual/auditory/kinesthetic (VAK) approach to learning (Huang et al., 2018; Riding 2001). VAK learning style framework is the most popular and extensively used categorization of the students' learning styles (Deborah et al., 2014; Khodabakhshzadeh et al., 2017). VAK is an abbreviation that stands for three key sensory modes of learning: Visual, Auditory, and Kinesthetic. This framework suggests that most learners can be divided into one of three preferred styles of learning, namely Visual, Auditory, and Kinesthetic (Fleming, 2001). Therefore, VAK learning style model is valuable for classifying the students' based on their interest in learning through visual, audio, and hands-on experiences (Deborah et al., 2014).

**Fig. 1:**
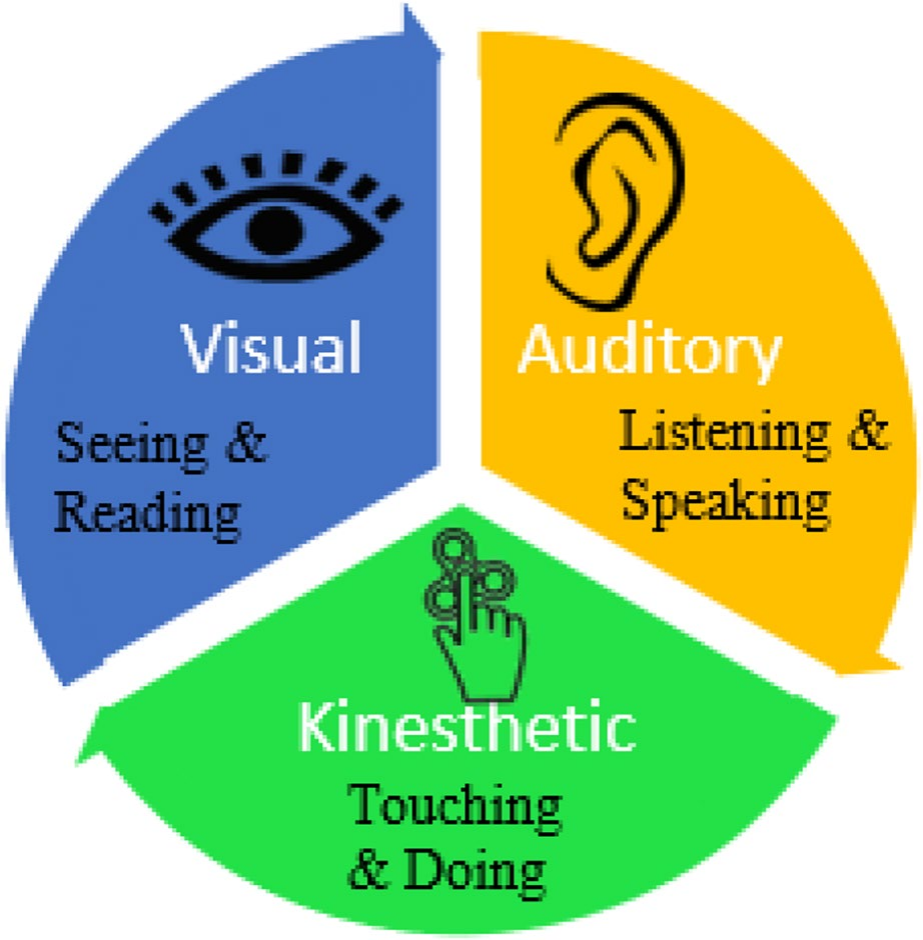
VAK Learning Style Framework

People with a Visual learning style learn greatest when information is delivered in the form of pictures, tables, charts, maps, or diagrams. On the other hand, auditory learners favor captivating information by listening. They feel comfortable when given instructions orally. Likewise, kinesthetic learners learn best by feeling and doing. They actually enjoy hands-on experience and like learning through practicals and group projects. Figure 1 depicts the VAK framework graphically.

### The Effects of Learning Style on the Learning Environment

The accurate identification of a students' learning style essentially helps teachers to assist the individual learner by designing and tweaking teaching techniques that meet the learner’s needs (Huang et al., 2018). Many researchers have described the positive effects of a teaching-learning process that is aligned with the learning style preferences of the learners (Altun & Serin, 2019; Azzi et al., 2020; Fatahi et al., 2016; Murray, 2004; Oxford et al., 1991; Rooney, 2018). Students' are expected to be more efficient and motivated in the learning environments that educators create by considering students’ learning styles (Altun & Serin, 2019). Likewise, Azzi et al. (2020) argue that knowing about students’ learning styles helps teachers personalize the learning to their students'. They highlight the importance of learning style identification as it aids in enhancing students’ learning boosting their motivation ,and condensing the time required for the learning.

Rooney (2018) argues that learning is boosted through the alignment of pedagogy and learning context. We, additionally, believe that the alignment between the pedagogy and students’ preferred learning style is also an important factor to consider while selecting a specific pedagogy. Morris (2004) also accentuates the relationship between the teaching approach and the learning style preference of the student as it is a factor in promoting the intrinsic motivation that boosts the learner’s curiosity, creates a desire to learn, and increases the level of interest to participate in the learning activity. On the contrary, if there is a misalignment between the teaching technique and the preferred learning style of the students, the conflict between the two will lead to a less-than-optimum or adverse learning environment for the student (Morris, 2004). By the same token, Oxford et al. (1991) concluded that style wars between teachers and learners result in student dissatisfaction with the learning process. They argued that learner dissatisfaction arising from a style conflict might cause negative feelings and behaviors ranging from mild discomfort and anxiety during the teaching-learning process to absenteeism and eventual withdrawal from the course altogether.

Learning styles’ influence is not limited to conventional learning environments only. Deborah et al. (2014) note that the psychological levels of the students in an E-learning environment are also significantly attributed to the learning styles of the learners involved in the learning process. They further argue that in many of the current E-learning models, the psychological level between the learners and the E-learning environment is not well aligned. The studies covering the topics related to students’ learning styles have predominantly addressed the identification of students’ learning style preferences (Huang et al., 2018); However, this area has not been explored in connection to the use of simulation-based teaching-learning processes. Consequently, it is the essence of such orientation that this study discovers the possibility of alignment between pedagogies of computer simulation and students’ preferred learning styles ,i.e., auditory, visual, and kinesthetic styles.

**Fig. 2:**
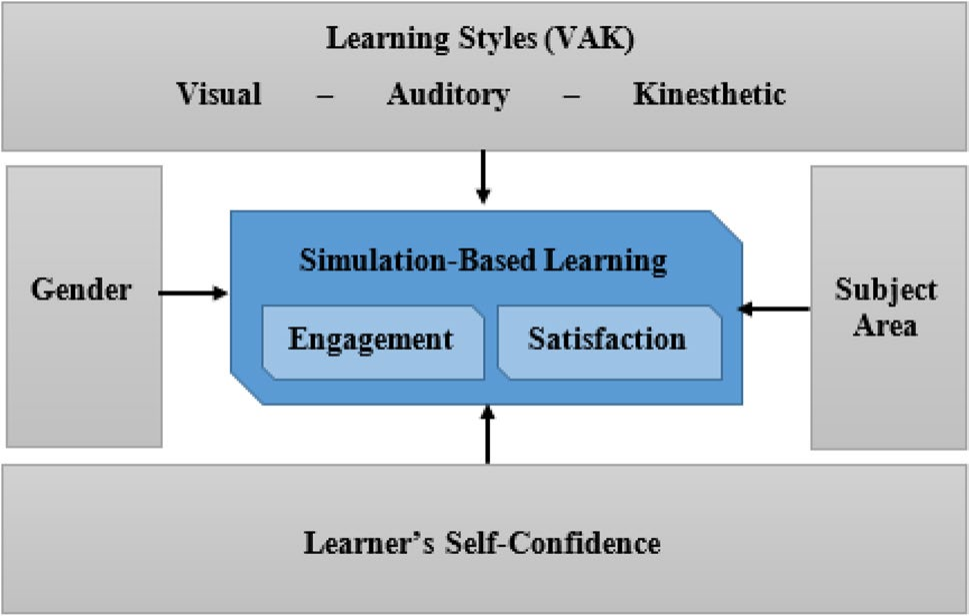
The Conceptual Framework of the Present Study

Although the use of simulations in teaching is a trending practice in the current time, our literature search disclosed that very few studies had been conducted that examined learners’ engagement and satisfaction with the use of simulations to enhance their learning experiences. Also, very little is known if students’ engagement and satisfaction with this teaching technique have significant differences in respect of their gender or the subject area of teaching. The authors found that the gender of students' influences the success or the failure of students' achievements and attitudes in science subjects and suggest that contexts and prevailing socio-cultural attitudes and beliefs surpass the impact of learning style (Almasri et al., 2021). Moreover, previous research has documented several factors that influence learner engagement (Khlaif et al., 2021). However, very little is known about the factors influencing student engagement with the learning environment that involves simulation-based activities. More specifically, adequate information is not available regarding the influence students’ self-confidence, and preferred learning style may have on their engagement and satisfaction with the simulation-based learning activities? Do the students' of a particular learning style get themselves engaged and feel more satisfied than the students of another learning style? The answers to these questions may help better understand the connections among these important factors involved in the use of computer simulation for teaching. Therefore, to fill these gaps in the literature, the purpose of our study is elicited in the format of a conceptual framework presented in Fig. 2, followed by specific research questions.

### Research Questions

Thus, our work is guided by the following research questions:

1. What are undergraduate students’ levels of Engagement and Satisfaction with the simulations for science learning?
2. Do undergraduate students’ Engagement and Satisfaction with the simulations for science learning significantly differ in respect of their gender or specific science courses (Physics, Chemistry, and Biology)?
3. Does learners’ self-confidence with the simulation positively influence their engagement and satisfaction with the simulation-supported learning activity?
4. Do students’ learning styles significantly influence their engagement and satisfaction with the simulation-supported learning activity?

## Methods

To meet the purpose of this survey study, a cross-sectional design was employed. A cross-sectional investigation encompasses observations of a sample, or cross-section, of a population or phenomenon that are made at one point in time (Babble, 2014).

### Sample and Context

The investigation took place in a large public university in a gulf country. The three science subjects ,i.e., physics, chemistry, and biology, were taught by the same teacher. The teacher used simulation-based teaching activities throughout the semester to teach the intended science concepts of the three subjects. Students from both genders were randomly assigned by the program administrative databases into classes. Simple random sampling was used to select the participants of the study.

Data collection occurred over two weeks after the semester ended. The researchers got approval from the Humanities & Social Sciences Research Ethics Committee (HSSREC) to carry out this study. In addition, the author received informed written consent from the participants. Questionnaires were responded to by 1034 students from three science subjects: Physics, Mathematics, and Biology. Due to COVID-19 social distance restriction, data were collected using hybrid mode. The hybrid mode of data collection (Soomro et al., 2019) helped to target a larger sample size. Fifty percent of responses (n=517) were collected in class ,while remaining data (n=517) were collected through online mode. There was a total of 536 (52%) male students, while 498 (48%) were female students. An almost equal number of students (33%) were taken from each science subject.

### The Simulation Resources Used

The instructor mainly used three open-access simulation resources to support the teaching of Physics, Chemistry, and Biology topics. The three resources included PHET Interactive Simulations (https://phet.colorado.edu/), SimPop (https://simpop.org/), and OLABS (http://www.olabs.edu.in/). The selected simulations are operated with mouse and keyboard controls only so they can be used on general-purpose digital devices such as computers, laptops, tablets, or smartphones ,and they did not require any special devices. Having a simple and straightforward user interface, all of the three simulation resources used were interactive in nature, allowing students to change some of the parameters in the program and observe what occurs as an effect.

**Fig. 3:**
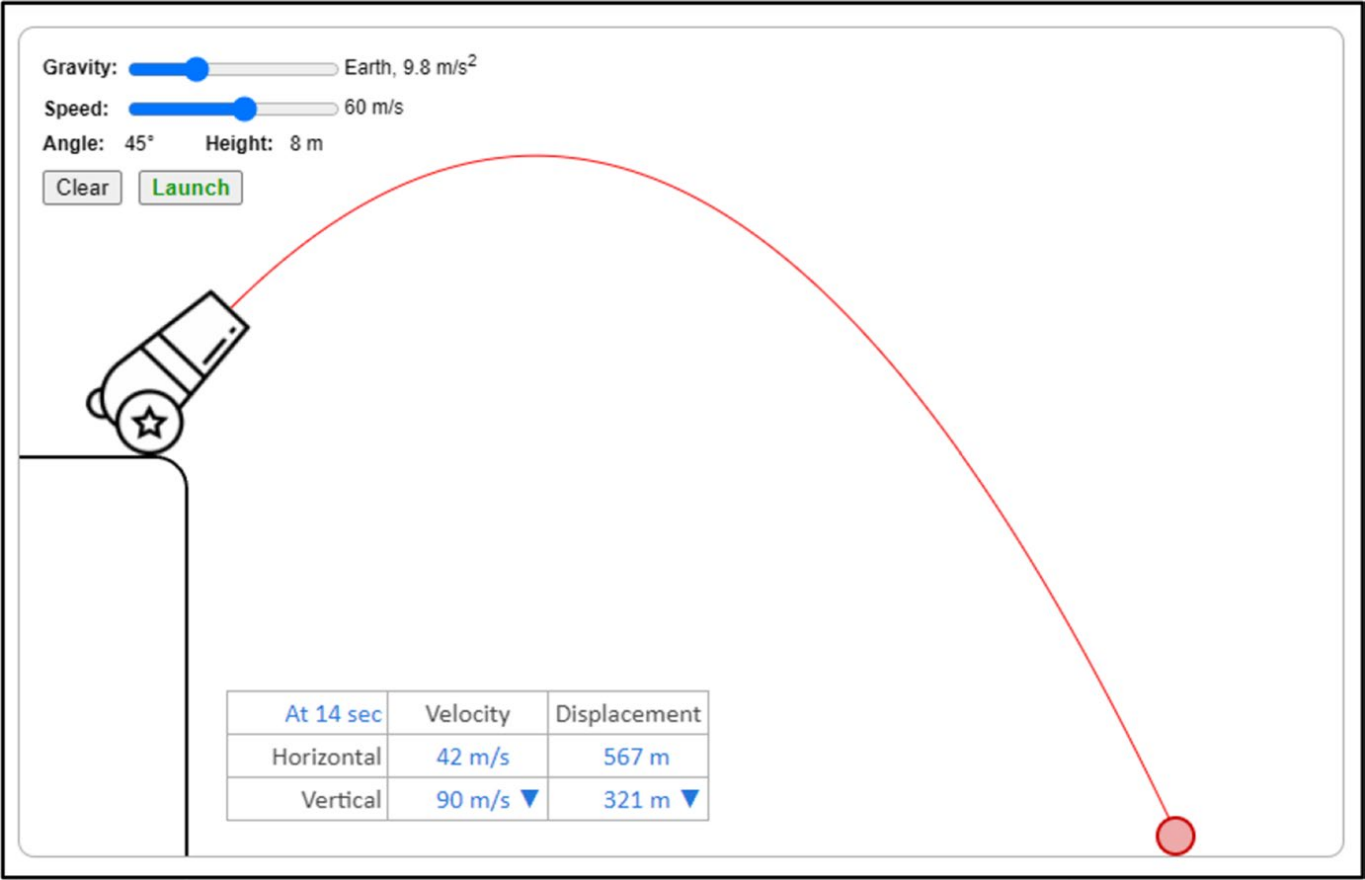
Teaching of Projectile Motion Using Simulations (https://simpop.org/)

**Fig. 4:**
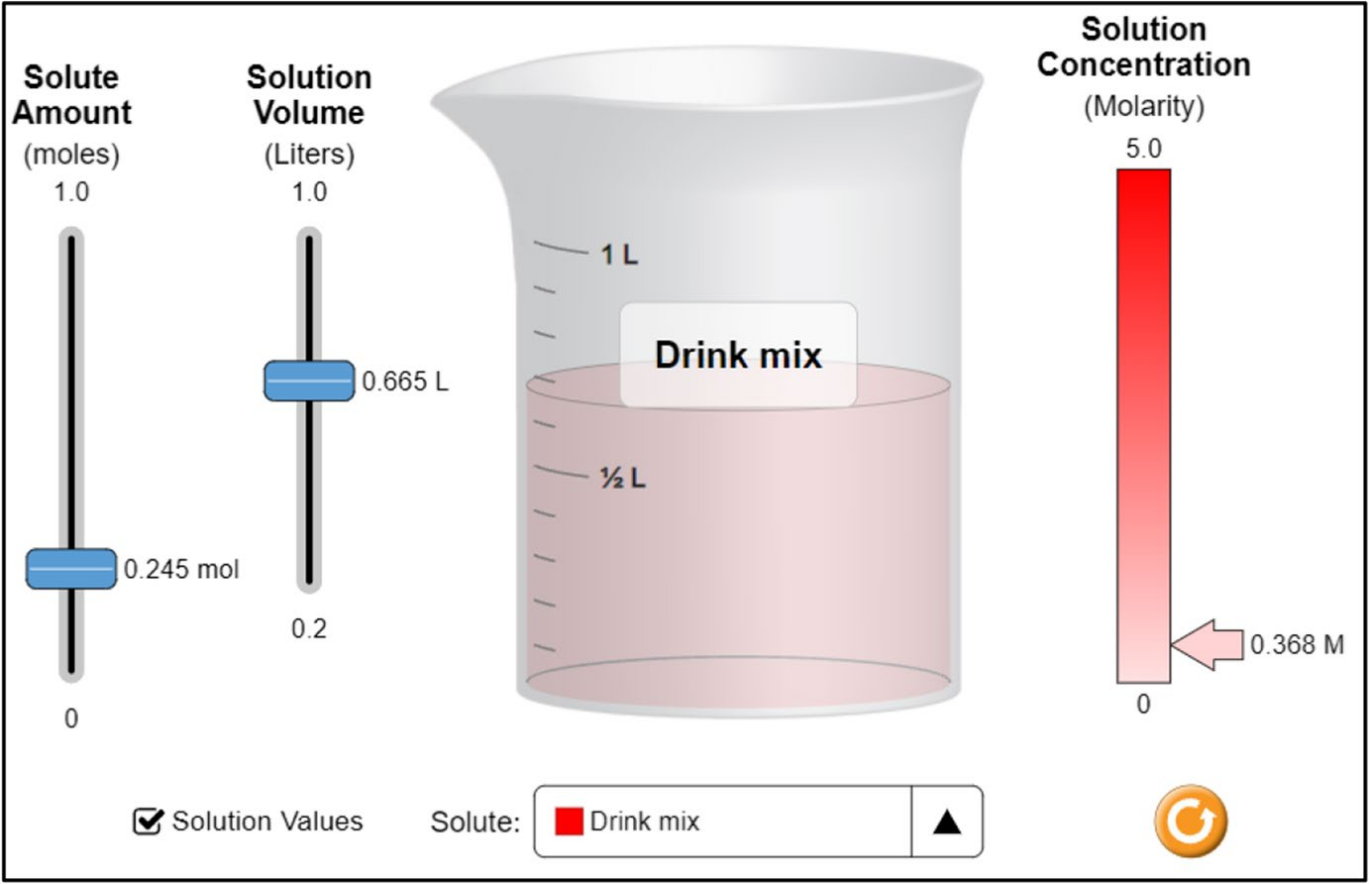
The Teaching of Molarity Using Simulations (https://phet.colorado.edu/)

**Fig. 5:**
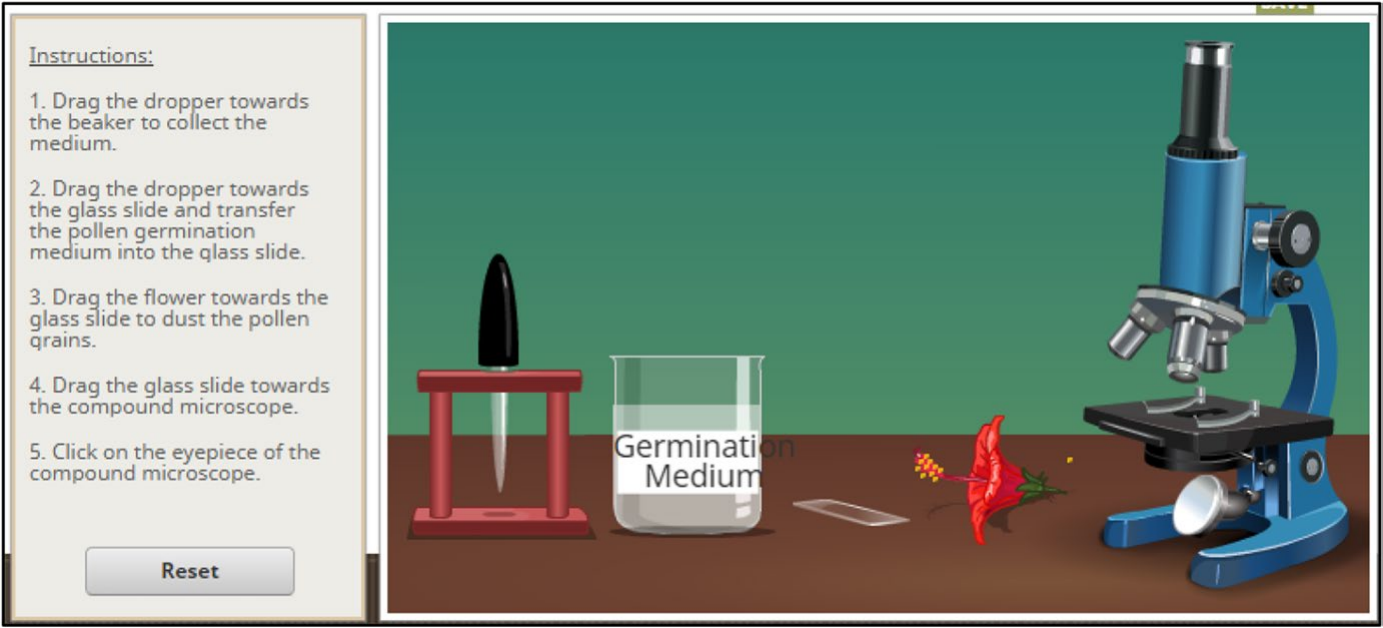
The Teaching of Pollan Germination Using Simulations (http://www.olabs.edu.in/)

The Physics simulations covered the topics such as Hooke's law, projectile motion, Coulomb's law, gravitational force, conservation of energy, and waves. As a sample, Fig. 3 depicts a computer simulation activity to teach projectile motion in a Physics class. The main topics for Chemistry simulation included atomic interaction, gas properties, molarity, molecular shape, and status of the matter. Figure 4 shows a simulation-based activity to teach molarity in a chemistry class. Similarly, the simulations for Biology teaching covered the topics including mitosis, human cell, plant cell, and simple staining and gram staining. Figure 5 shows an activity to teach students about pollen germination through a computer simulation.

### Research Instrument

The data for the present study was collected through a close-ended questionnaire which consisted mainly of four sections. The first section targeted recording participants' demographic information such as their age, gender, and subject area. The second section of the survey measured participants’ engagement with the simulation-based learning they had recently experienced in science courses. This section, adapted from (Lindgren et al., 2016), consisted of 10 items formatted on a 5-point Likert scale (1=Strongly disagree, 2=Disagree, 3=Neutral, 4=Agree, and 5=Strongly agree). These items were intended to measure the magnitude of cognitive and perceptual involvement learners felt in their experience with the simulation-based learning activities.

The third section focused on participants’ satisfaction and self-confidence with the learning process. To meet the intent of this section, the Student Satisfaction and Self-Confidence in Learning Scale (Jefferies & Rizzolo, 2006) was employed. This section consisted of 12 items. Lastly, participants’ preference of their learning style was assessed using the Learning Style Questionnaire (O’Brien, 1989). Formatted on a 3-point Likert scale (1=Never applies to me, 2= Sometimes applies to me, and 3= Often applies to me), the scale consisted of 30 items out of which 10 items focused on a specific learning style including visual, auditory, and kinesthetic learning styles.

In order to confirm the validity and feasibility of the three scales to be used in the research context of the present study, we sought expert opinion from the three researchers/university faculty in the field. Moreover, the questionnaire was pilot tested with a small group of potential participants. The reliability analysis was conducted using the internal consistency method through Cronbach's Alpha coefficients. The internal consistency reliability coefficients were ≥ .80 for all scales for both male and female subgroups except for a few cases like self-confidence for female groups which is low .65. Overall reliability coefficients were above .80, which shows a very good reliability coefficient. All scales’ reliability coefficients were from 0.80 to 0.90 range which is over the recommended level of 0.7 (Roldán & Sánchez-Franco, 2012). Scale-wise reliability coefficients for all samples and male and female subgroups are reported in Table 1.

**Table 1:** Reliability Analysis of Instruments (N=1034)

Scale	No# of items	Male	Female	Overall
Engagement	10	.83	.77	.82
Satisfaction	5	.83	.80	.82
Self Confidence	7	.78	.65	.73
Visual Learning	10	.86	.89	.88
Auditory Learning	10	.89	.91	.90
Kinaesthetic Learning	10	.90	.91	.91
Overall Learning Style	30	.94	.96	.95

### Data Analysis

Considering the nature of the study and the close-ended nature of questions asked in the questionnaire, all data were analyzed quantitatively using Statistical Package for the Social Sciences (SPSS, version 21). Descriptive statistics ,including frequency, mean, and standard deviation were calculated and inferential tests including students’ t-test, ANOVA, and linear regressions, were conducted. Statistical significance was set at p<0.05.

## Results

The primary aim of this study was to examine tertiary students’ simulation-based learning experiences for science learning. We focused on examining if their engagement and satisfaction with the learning process were significantly different in respect of their gender and subject area. Moreover, we were interested to examine if learners’ self-confidence and their preferred learning styles influence their engagement and satisfaction with the simulation-based learning experiences. The results of specific research questions are presented in the following:

- R.Q. # 1 - What are undergraduate students’ levels of Engagement and Satisfaction with the simulations for science learning?

The first research question was related to assessing students’ levels of engagement and satisfaction with the simulation-based activities for science learning. Table 2 shows the overall students’ level of engagement and satisfaction according to their science subject. Descriptive analysis indicated that students’ level of engagement and satisfaction towards the simulated science learning activities. A higher mean value indicates a higher level of agreement toward stimulation for science learning. According to Table 2, students’ engagement is a relatively higher indicator to simulate for science learning ,especially for students' from biology subject groups. In general, students' from all three subject groups reported that they were highly engaged and felt highly satisfied with the simulation-based activities to support their learning experience for physics, chemistry, and biology courses.

**Table 2:** Mean and Standard Deviation of Students level of Engagement and Satisfaction for each Subject Group

Subject groups		Engagement	Satisfaction
Physics (N=346)	M	3.41	3.14
	SD	1.33	1.61
Chemistry (N=346)	M	3.50	2.80
	SD	1.04	1.50
Biology (N=342)	M	3.81	3.46
	SD	0.90	1.36
Overall (N=1034)	M	3.57	3.13
	SD	1.12	1.52

- **RQ # 2 - Do undergraduate students’ Engagement and Satisfaction with the simulations for science learning significantly differ in respect of their gender and specific science courses (Physics, Chemistry, and Biology)?**

The second research question was focused to examine significant differences in learners’ engagement and satisfaction with the simulations for science learning based on their gender, and subject areas i.e., physics, chemistry, and biology.

The results of an Independent t-test indicated that there is a significant difference between males and females in their level of engagement towards simulation in science learning, *t*(1032) = 8.55, *p*<.001, partial eta squared = .05. In addition, the result also indicated a significant difference found between males and females in their level of satisfaction with the computer-based simulations in the science subjects, *t*(1032) = 3.14, *p*=.002, partial eta squared=.02. Moreover, female students' (*M*=3.87, *SD*=0.94) reported a significantly higher level of engagement with the simulations in science subjects than male students' (*M*=3.30, *SD*=1.19). As far as the level of satisfaction is a concern, both male and female students' did not report any major difference. Females reported a relatively higher level of satisfaction than male students' with simulations in science subjects (see Table 3).

**Table 3:** Independent t-test results between males and females in three study variables

Variables	Gender	N	M	SD	t	Sig	Effect size
Engagement	Male	536	3.30	1.19	8.55	<.001	0.05 (Medium)
	Female	498	3.87	0.94			
Satisfaction	Male	536	2.99	1.57	3.14	0.002	0.02 (Small)
	Female	498	3.29	1.44			

The Analysis of variance (ANOVA) result indicated that there is a significant difference in students’ level of engagement, *F*(2, 1031) = 13.12, *p*<.001, partial eta squared = .025 (medium effect size) and their level of satisfaction, *F*(2, 1031) = 16.76, *p<*.001, partial eta squared = .031 (medium effect size) with simulation in science learning based on students' course subjects. Moreover, posthoc analysis revealed that Biology students have a significantly higher level of engagement with simulation in science learning than Chemistry and Physics course students'. Similar trends of results were found in students’ level of satisfaction. Biology course students' have a significantly higher sense of satisfaction than students from Physics and chemistry groups (see Table 4 for details).

**Table 4:** Analysis of Variance results for three study variables based on Science Subject groups

		N	M	SD	F	Sig	Partial Eta Squared
Engagement	Physics	346	3.41	1.32	13.12	<0.001	.025
	Chemistry	346	3.50	1.04			
	Biology	342	3.81	0.90			
Satisfaction	Physics	346	3.14	1.61	16.76	<0.001	.031
	Chemistry	346	2.80	1.50			
	Biology	342	3.46	1.36			

**Fig. 6:**
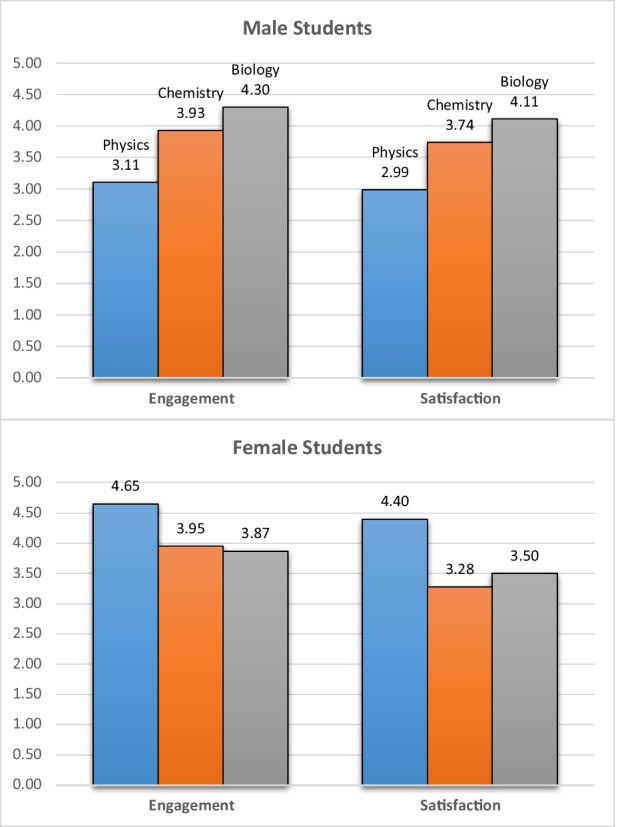
Male and Female Students level of Engagement and Satisfaction according to Science Major Subject

Furthermore, Fig. 6 shows gender-wise and subject-wise students’ level of engagement, satisfaction, and self-confidence with simulation for science learning.

Female students have a relatively higher level of engagement in science learning with simulation than male students, especially in Physics subjects. Female students from Physics subject also have a higher level of satisfaction than male students of the same subject. In general, descriptive results show that female students have a relatively higher level of engagement and satisfaction than male students regardless of which science subject they belong to.

### Does learners’ self-confidence with the simulation positively influence their engagement and satisfaction with the simulation-supported learning activity?

Regression analysis indicated that there is a significant and positive influence of students' self-confidence on their level of engagement with the simulation-supported learning activity, *F*(1, 534)=140.56, *p*<.001, R-squared = .21 for male students and, *F*(1, 496)=10.15, *p*=.002, R-squared = .14 for female students. The corresponding beta coefficient values for males and females were .41 and .12 respectively. Thus, it can be concluded that males’ self-confidence has a relatively higher influence on their engagement as compared to females’ self-confidence’s influence on their engagement.

Similarly, another regression analysis showed that there is a significant and positive influence of students' self-confidence on their satisfaction with the simulation-supported learning activity, *F*(1, 534)=76.06, *p*<.001, R-squared = .13 for male students and, *F*(1, 496)=8.85, *p*=.003, R-squared = .02 for female students. The corresponding beta coefficient values for males and females were .42 and .17 respectively. Similar trends of results were found; male students’ self-confidence have higher impact on their satisfaction as compared to female’s self-confidence impact on their satisfaction with the simulation-supported science learning activities.

### Do students’ learning styles significantly influence their engagement and satisfaction with the simulation-supported learning activity?

Our final research question was based on students learning style and its effect on students’ level of engagement and satisfaction with the simulation-supported science learnings. Results indicated that there is a significant positive influence of learning styles on students’ engagement *F*(3, 1029)= 15.35, *p*<.001, and satisfaction, *F*(3, 1029)= 8.97, *p*<.001. Three learning styles were measured: visual, auditory, kinesthetic. Simple regression analyses were performed to assess the influence of each learning style on student engagement and satisfaction. As shown in Table 5, all three types of learning styles significantly increase student engagement and satisfaction with the simulation-supported learning activities. Kinesthesis learning style has a relatively higher influence on student engagement and satisfaction as compared to the other two learning styles. Moreover, these three learning styles have more influence on student engagement as compared to their satisfaction while learning science through simulation-supported activities.

**Table 5:** Regression Analysis of three learning Styles on Student Engagement and Motivation

Learning Styles	Engagement				Satisfaction			
	R-square	F	Sig	Beta	R-square	F	Sig	Beta
Visual	.021	22.27	<.001	0.032	.012	12.94	<.001	0.030
Auditory	.020	21.19	<.001	.028	.017	17.49	<.001	0.035
Kinaesthetic	.043	46.04	<.001	0.040	.024	25.61	<.001	0.041

## Discussions

This study intended to examine students’ engagement, self-confidence, and satisfaction with the computer simulations for science learning and explore if these variables significantly differ in respect of their demographic characteristics. Another primary intention of the study was to see the possible influence of learning styles on learners’ engagement, self-confidence, and satisfaction with the simulation-based learning process.

First, the study empirically showed that tertiary students were highly engaged and satisfied with the simulation-based activities to support their learning of science subjects including physics, chemistry, and biology. Although this study did not focus on student achievement explicitly, learners’ engagement reflects their emotional, behavioral, and cognitive attachment with the learning process which has a direct impact on student success and achievement (Farrell & Brunton, 2020; Almasri et al.,2021). Since engagement is a reflector of the level of learners’ involvement and interaction with the learning environment (Bond, 2020; Khlaif et al., 2021; Almasri et al.,2021), students’ high engagement and satisfaction with this innovative pedagogical approach are very encouraging to accept the idea of using computer simulations for the teaching of science subjects such as physics, chemistry, and biology in higher education settings. Particularly, this finding supports the premise that the use of computer simulations is a great source of effectively anchoring the learners with the learning process.

The empirical results from this work provided interesting evidence for gender and subject-based significant differences in learners’ engagement with the simulation activities. The findings suggested that female students were on the higher side of engagement and satisfaction with simulation-based learning in comparison to their male counterparts. Also, our study revealed that students of the biology group reported having higher engagement as well as satisfaction with the learning process in comparison to the students of physics and chemistry groups. However, the effect size was of both the gender as well as subject-based differences was very small. Moreover, it was found that students’ self-confidence with the learning process was a significant positive predictor of their engagement and satisfaction with simulation-based learning. We did not find previous literature that could provide evidence in favor or against these findings.

Finally, the results also revealed that students' learning styles, particularly the kinesthetic style were a significant positive predictor of their engagement and satisfaction with the use of computer simulations for science learning. These findings are in agreement with the previous research that shows learners' engagement is positively influenced by kinesthetically-enhanced learning activities with digital technologies in a variety of contexts (Anastopoulou et al., 2011; Lindgren et al., 2016). Students with kinesthetic learning styles hate sitting idle and like activities that require them to do something. These learners prefer to touch, feel, experience, and manipulate. They attack problems by doing and regularly selecting the solution that requires the greatest activity. The positive connection between the kinesthetic style and learners’ engagement as well as their satisfaction with the simulation-based learning must have arisen from the inherited features of computer simulations that stress the learner to be an active agent in the process of knowledge construction and let learners effortlessly manipulate objects, generate novel representations, carry out experiments to test hypotheses and tentative ideas (Wang et al., 2010). Although the new generations of students and instructors in higher education widely accept simulation-based learning in general, the finding from this study implies that this particular pedagogical method might not be well received by the learners who have different learning styles such as auditory and visual (Juan et al., 2017).

### Limitations and Future Research Opportunities

Some limitations need to be acknowledged regarding the present research. The major limitation lies in the fact that the scope of the current study was limited to students’ experiences with simulation-based teaching at the tertiary level in a university from a Gulf country. To expand the understanding on this topic, research is required to back the findings of the present study and further confirm the impact of learning styles on the effectiveness of the use of computer simulations to teach science concepts in different settings such as school and college levels. Secondly, while assessing participants’ engagements and satisfaction with this pedagogical approach, we did not consider the levels of their digital skills and general usage of ICT. Since the individuals having a higher level of ICT usage are more likely to adopt digital technologies in their teaching-learning practices (Soomro et al., 2020), the students’ engagement and satisfaction with simulations for learning, reported in this study, might have been influenced by their general ICT skills and usage. Furthermore, Studies based on true-experimental designs can be helpful to generalize the findings with high confidence. Moreover, literature examining teacher attitudes toward simulation use in teaching is insufficient (Lee et al., 2021). Besides students’ engagement and satisfaction with the simulation-based teaching practices, teacher attitudes and their experiences of using simulation to support their teaching of science subjects is another dimension that needs further investigation.

## Conclusion

This study explored tertiary students’ learning experiences with the computer simulations for science subjects. Our study shows that the pedagogical use of computer simulations is a great way for teaching science subjects including physics, chemistry, and biology. This approach of teaching science subjects is helpful to educators gaining a level of students’ engagement with the learning process. Furthermore, the study revealed gender and subject-based differences in students’ level of engagement and satisfaction. Our work also contributes to the argument regarding the role of learning style in learners’ engagement and satisfaction with simulation-based learning. It is concluded that learning style is a significant predictor of learners’ engagement and satisfaction. Specifically, students who prefer learning by doing (the kinesthetic style) were highly engaged with the learning through interactive computer simulations.

## Data Availability

Data is available upon request from the author.
